# Use of Permanent Markers for Intraoperative Marking in Body Contouring Surgery: An Innovative Technique

**DOI:** 10.29252/wjps.7.3.387

**Published:** 2018-09

**Authors:** Shabeer Ahmad Wani, Loai Abdullah Al Salmi, Ovais Habib, Mir Uzair Ul Haq

**Affiliations:** 1 Section of Plastic Surgery, King Fahad Medical City, Riyadh, Saudi Arabia;; 2 Medical intern SKIMS Medical College, Srinagar, Kashmir, India

**Keywords:** Sharpie permanent, Marker, Intraoperative Marking

**DEAR** **EDITOR**

Body contouring surgery after massive weight loss is becoming very common all over the world and more so in our part of world. The contouring procedures need an extensive preoperative planning and correct marking of the incisions and areas of resection. The marking fades sometimes after scrubbing the patient and use of tumescent fluids. Intraoperative precise marking can be done by use of sterile surgical markers (Figure 1). The scarcity of ink in these markers is frustrating and sometimes we need to open multiple markers, which is not cost effective. Another alternative is to use methylene blue with a Q-tip applicator. The marking by this method is not precise. We have been using Sharpie (Atlanta, Georgia, USA) colored permanent markers in a sterile pouch made from draping sheets. Circulating nurse decontaminates the outer surface of pen with an alcohol swab and cap of pen is removed. 

**Fig. 1:**
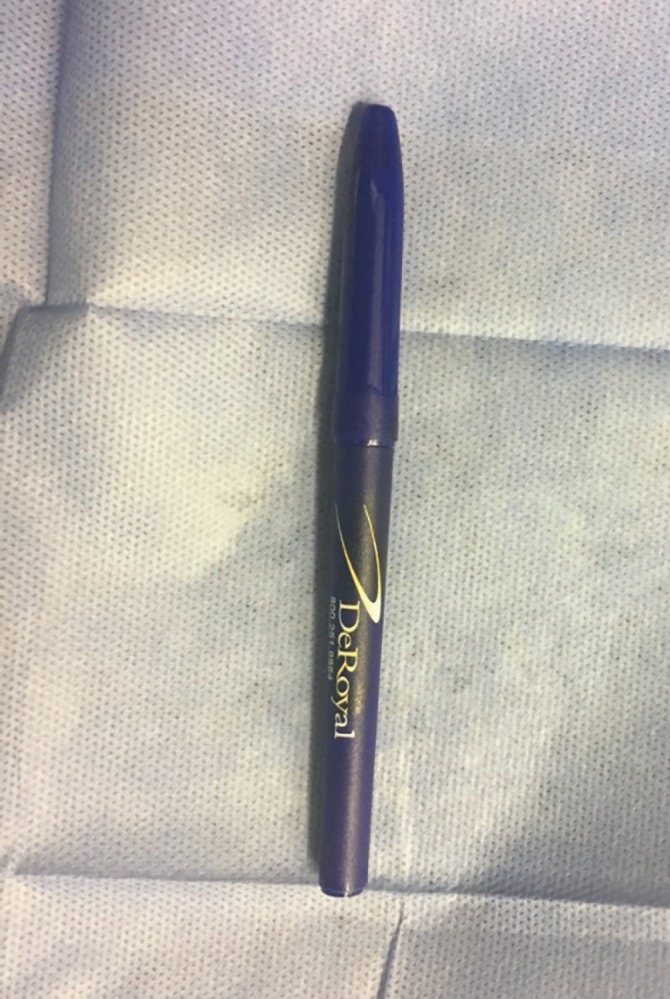
Intraoperative precise marking done by use of sterile surgical markers

The circulating nurse hands over the pen to scrub nurse who puts it in a pouch made from sterile drape. Putting steri strips over it tightens the pouch (Figure 2). The writing tip of this marker is sterile by itself as it contains isopropyl alcohol. The antibacterial action of the writing tip has been proven in a research done at university of Alberta.^1^ At the end of the procedure the pen is removed from the pouch and recapped, and can be used in this way for multiple times. This method is cost effective, and we have an option of using multiple colors. Moreover, the Sharpie markers have ink, which dries on the surface of patient instantaneously and marks don’t fade. We did not notice any infection in these patients, which could have been related to use of these markers.

**Fig. 2:**
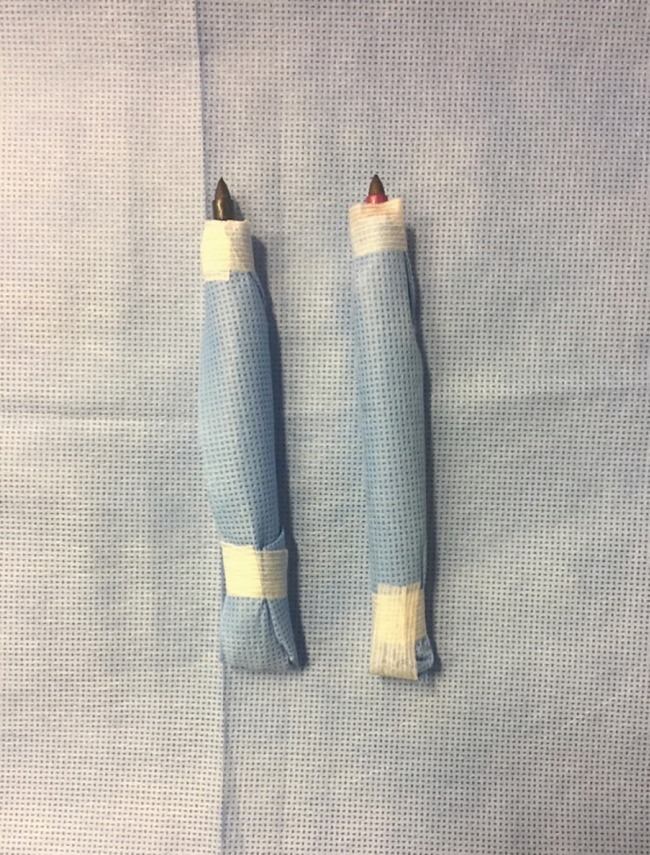
The circulating nurse hands over the pen to scrub nurse who puts it in a pouch made from sterile drape. Putting steri strips over it tightens the pouch

## CONFLICT OF INTEREST

The authors declare no conflict of interest.
